# *In silico* discovery of terpenoid metabolism in *Cannabis sativa*

**DOI:** 10.12688/f1000research.10778.1

**Published:** 2017-02-06

**Authors:** Luca Massimino

**Affiliations:** 1 Molecular Oncology Unit, San Gerardo Hospital, Monza, Italy

**Keywords:** terpenoid metabolism, cannabis sativa, in silico gene expression, evolutionarily conserved genes

## Abstract

Due to their efficacy, cannabis based therapies are currently being prescribed for the treatment of many different medical conditions. Interestingly, treatments based on the use of cannabis flowers or their derivatives have been shown to be very effective, while therapies based on drugs containing THC alone lack therapeutic value and lead to increased side effects, likely resulting from the absence of other pivotal entourage compounds found in the Phyto-complex. Among these compounds are terpenoids, which are not produced exclusively by cannabis plants, so other plant species must share many of the enzymes involved in their metabolism. In the present work, 23,630 transcripts from the canSat3 reference transcriptome were scanned for evolutionarily conserved protein domains and annotated in accordance with their predicted molecular functions. A total of 215 evolutionarily conserved genes encoding enzymes presumably involved in terpenoid metabolism are described, together with their expression profiles in different cannabis plant tissues at different developmental stages. The resource presented here will aid future investigations on terpenoid metabolism in *Cannabis sativa*.

## Introduction

Due to its astonishing efficacy ^1^, nowadays cannabis is prescribed by physicians for the treatment of neurological, psychiatric, immunological, cardiovascular, gastrointestinal, and oncological conditions ^2– 7^. Although therapies based on the use of cannabis flowers or their derivatives are recognized to be very effective, treatments centered on drugs containing Δ ^9^-tetrahydrocannabinol (THC) alone lack efficacy and lead to increased side effects ^8, 9^. This discrepancy seems to result from the absence of the synergistic effects of additional pivotal compounds found in the Phyto-complex, the so-called entourage effect ^10^. Among these molecules are other cannabinoids and terpenoids, which are thought to play major roles in the modulation of THC ^11^.

Terpenes are small hydrocarbon (isoprenoid) molecules classified as either as monoterpenes, sesquiterpenes, diterpenes or carotenoids, depending on the number of isoprene units (C _5_) used to synthetize them. Terpenoids are small lipids derived from terpenes, often accompanied by a strong odor useful for the plants to protect themselves against possible predators ^12^. Terpenoids not only have important functions when working in concert with cannabinoids; they have been widely investigated in many different plant species and are being exploited as anti-fungal, anti-bacterial, anti-oxidant, anti-inflammatory, anti-stress, anti-cancer and analgesic agents ^13– 18^. However, whilst the gene networks controlling the biosynthesis of cannabinoids and their precursors have been extensively studied ^19– 22^, the biosynthetic pathway of terpenoid molecules in *Cannabis sativa* is only recently being elucidated. Only two genes have been characterized, one encoding (-)-limonene synthase, the other (+)-α-pinene synthase ^23^, two enzymes responsible for the conversion of geranyl pyrophosphate into limonene and pinene, respectively ^24, 25^. Remarkably, while cannabinoids are only found in cannabis plants, terpenoids are also produced by a variety of other plant species, so they must share many of the enzymes involved in their metabolism.

In the present work, evolutionarily conserved genes encoding enzymes predicted to be involved in terpenoid metabolism have been identified within the transcripts of the canSat3 reference transcriptome of *Cannabis sativa* ^21^. Moreover, by taking advantage of available gene expression data ^26^, gene expression profiling of these enzymes was performed in cannabis plant tissue at different developmental stages. The data note presented here will provide researchers with a corollary of candidate genes that will considerably accelerate future investigations on terpenoid metabolism in *Cannabis sativa*.

## Material and methods

### Analysis of evolutionarily conserved transcripts

Cannabis sativa transcript sequences (n=23,630) taken from the canSat3 genome assembly ( http://genome.ccbr.utoronto.ca/) ^21^ were annotated with Blast2GO 4.0.7 ^27^ using NCBI blastx and InterProScan databases. Terpene metabolism related genes were selected if found to be present in datasets downstream of the “terpene metabolic process” gene ontology category from the AmiGO 2 repository ( GO:0042214), including the carotene metabolic process ( GO:0016119), ent-kaurene metabolic process ( GO:0033331), ent-pimara-8(14),15-diene metabolic process ( GO:1901539), isoprene metabolic process ( GO:0043611), miltiradiene metabolic process ( GO:1901944), monoterpene metabolic process ( GO:0043692), sesquarterpene metabolic process ( GO:1903192), sesquiterpene metabolic process ( GO:0051761), terpene biosynthetic process ( GO:0046246), and terpene catabolic process ( GO:0046247) ^28^.

### Gene expression analysis

Gene expression profiles from cannabis plant tissue at different developmental stages were downloaded from the NCBI GEO repository ( https://www.ncbi.nlm.nih.gov/geo/). Gene expression heatmaps and unsupervised hierarchical clustering were performed with GENE-E 3.0.213 ^29^.

## Results

**Figure 1.**
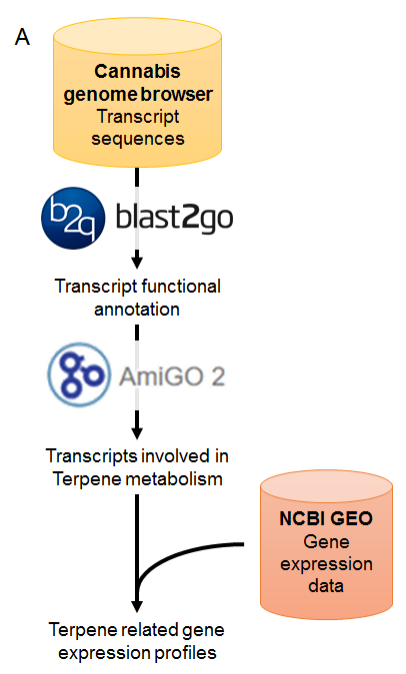
Workflow analysis. Schematic of bioinformatics pipeline utilized in this work. *Cannabis sativa* transcript sequences were taken from the canSat3 reference genome, functionally annotated with Blast2GO 4.0.7, filtered for terpenoid metabolism categories (AmiGO 2), and integrated with gene expression data downloaded from NCBI.

**Figure 2.**
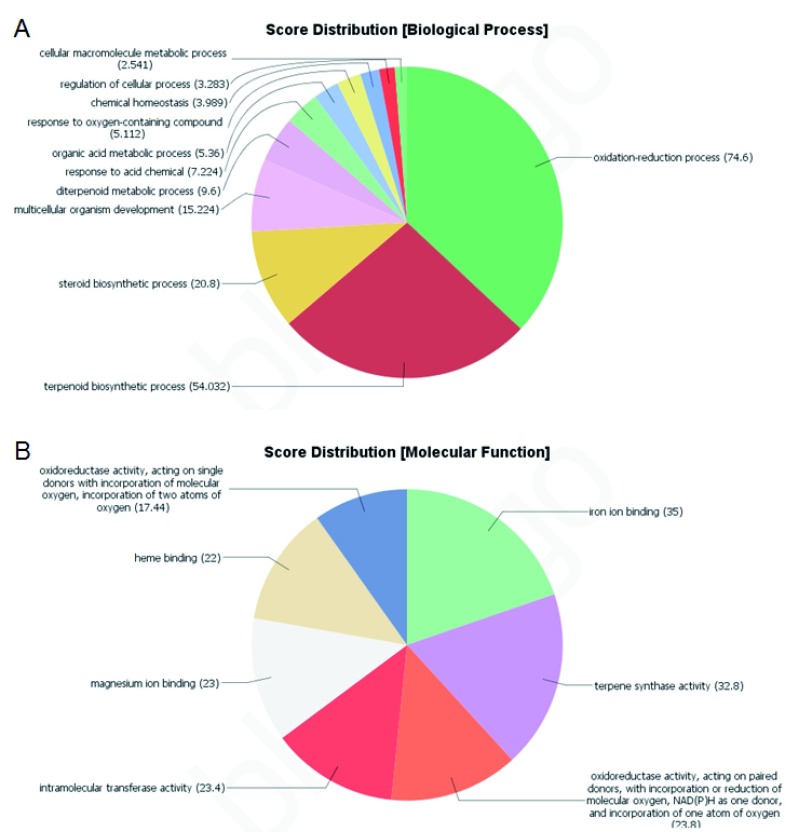
Gene ontology analysis of putative terpenoid metabolism related transcripts. Functional enrichment analysis of putative terpenoid metabolism related transcripts taken from the canSat3 reference genome. Enrichment for terpene biosynthesis and catabolism is shown for Biological Process ( **A**) and Molecular function ( **B**) Gene ontology categories.

Although the *Cannabis sativa* reference genome and transcriptome has been publicly released ^21^, only a few genes have been characterized and surveyed for their molecular functions. To define the possible roles of these genes, 40,197 canSat3 transcript sequences were downloaded from the cannabis genome browser ( http://genome.ccbr.utoronto.ca/), translated *in silico*, and scanned for evolutionarily conserved protein domains for functional annotation ( Figure 1). To identify the genes presumably playing a role in terpenoid metabolism, annotated transcripts were filtered for gene ontology (GO) categories involved in terpene biosynthesis and catabolism using the AmiGO 2 reference database. A total of 288 transcripts representing 215 different genes were predicted to be involved in the metabolism of bisabolene, cadinene, carotene, copaene, ent-kaurene, farnesol, geraniol, germacrene, lycopene, limonene, myrcene, phytoene, pinene, squalene, and others ( Supplementary table 1). Functional characterization of this subset confirmed an enrichment for GO categories involved in different terpene biosynthetic and catabolic processes ( Figure 2).

**Figure 3.**
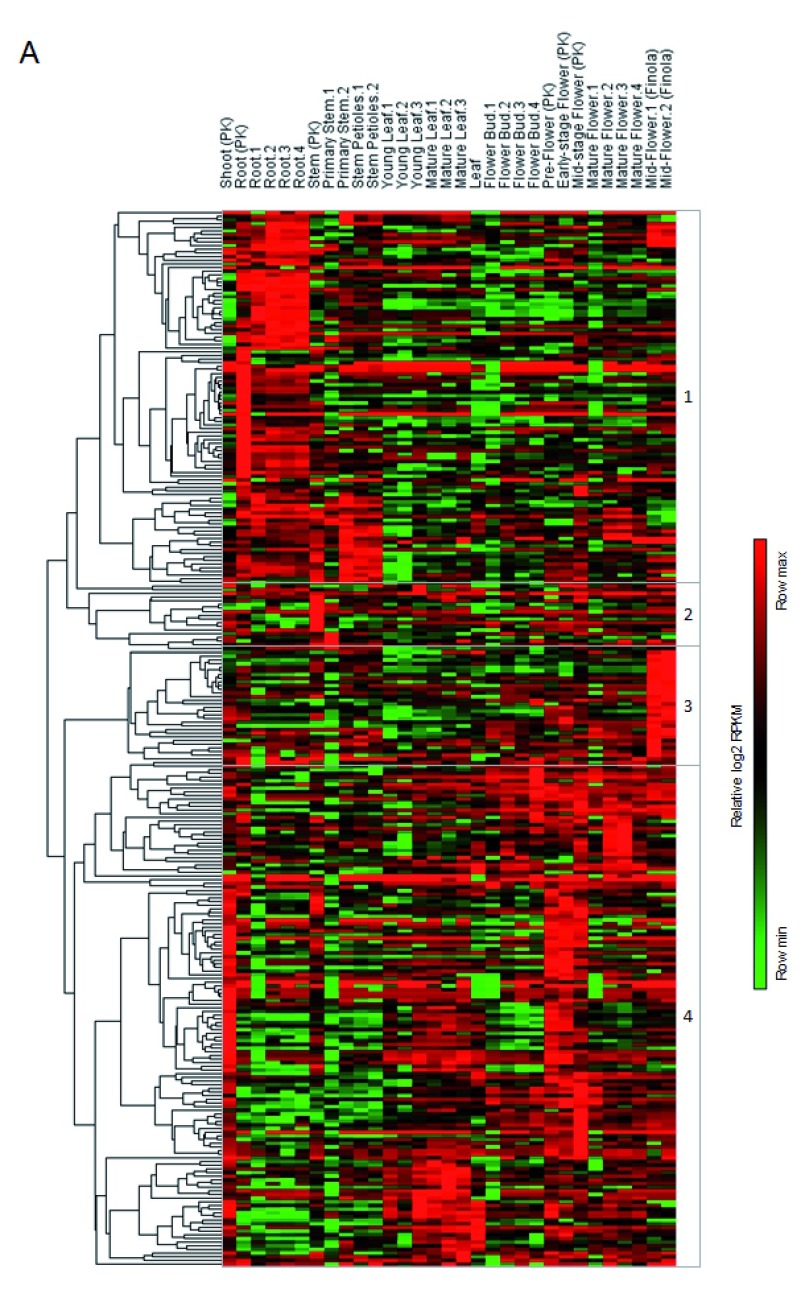
Gene expression analysis of terpenoid metabolism related genes. Heatmap showing relative expression values (log2 RPKM) of putative terpenoid metabolism related genes from cannabis plant tissue taken at different developmental stages (shoot, root, stem, young and mature leaf, early-, mid- and mature-stage flower). Five gene clusters were defined in accordance to unsupervised hierarchical clustering.

Terpenoids are produced by several plants species and in several types of plant tissue as defense against predators ^30^. Similar to other biological compounds, their abundance directly correlates with the expression levels of the enzymes involved in their metabolism. To this end, gene expression analysis of genes likely to be involved in terpenoid metabolism was performed using previously published datasets ( Figure 3; Supplementary table 2) ^26^. Notably, unsupervised hierarchical clustering identified four gene clusters. Cluster 1 genes display high expression in roots and stems; cluster 3 genes in hemp flowers; cluster 4 genes in leaves and flowers. Cluster 2 genes were constitutively expressed in all tissues. These results highlight which enzymes are expressed by specific tissues and will provide a strong rationale for further investigations on the molecular basis of terpenoid metabolism.

## Discussion

The active principles inside plants have been exploited by humans for centuries, with *Cannabis sativa* being one of the oldest ever used for medicinal purposes ^31^. Surprisingly, contrary to whole plant extracts, medicinal products containing exclusively THC have been found to lack efficacy and lead to unbearable side effects ^8, 9^. These results arise from the fact that these products lack other important co-factors typically found in the Phyto-complex, such as terpenoids and other cannabinoids ^10^ that contribute to the synergistic effects seen with whole plant extracts.

While genes involved in cannabinoid biosynthesis have been widely investigated ^19– 22^, the gene network controlling terpenoid metabolism is only recently being elucidated, with genes encoding (-)-limonene synthase and (+)-α-pinene synthase being the only two characterized ^23^. To this end, *Cannabis sativa* transcripts ^21^ have been scanned for evolutionarily conserved protein domains and annotated according to their presumptive molecular function. As a result, 215 evolutionarily conserved genes were predicted to be involved in terpenoid metabolism. Furthermore, *in silico* gene expression profiling ^26^ of these enzymes in cannabis plant tissue at different developmental stages highlighted different gene clusters with peculiar expression patterns. For instance, cluster 3 genes ( Figure 3) displayed high expression specifically in hemp flowers, which could be of great interest as different cannabis strains harbor different entourage effects.

Since the current cannabis reference transcriptome is still at preliminary stages ^21^, it is very likely that false negatives have caused important transcripts to still be missing. For example, the two genes encoding for (-)-limonene synthase and (+)-α-pinene synthase ^23^ align on the same transcript predicted to encode for Myrcene synthase ( PK25781.1 in Supplementary table 1), and therefore cannot be discriminated. Unfortunately, to overcome this issue at whole genome level we need the complete version of the reference transcriptome to be available. Until that time, researchers are forced to validate single transcripts with classic low-throughput technology, such as molecular cloning followed by Sanger sequencing.

Nevertheless, the data presented here will ease future investigations on terpenoid metabolism in *Cannabis sativa* by providing researchers with a collection of candidate genes. For instance, one of these genes was predicted to encode for β-bisabolene synthase ( PK05069.1 in Supplementary table 1). Bisabolene is being used as an antimicrobial agent ^32^, as well as a biofuel ^33^. However, prior to this report nothing was known about the gene network controlling its metabolism in *Cannabis sativa*. As soon as future studies will integrate gene expression data with chemical analysis, the complete molecular scenario underlying terpenoid metabolism will be revealed.

## Data availability

The data referenced by this article are under copyright with the following copyright statement: Copyright: © 2017 Massimino L

Processed gene expression data can be found in the NCBI GEO repository ( https://www.ncbi.nlm.nih.gov/geo/) with accession number GSE93201.

## Supplementary material

**Supplementary table 1. Evolutionarily conserved terpenoid metabolism transcripts**

List of putative terpenoid metabolism genes obtained with Blast2GO.

.

**Supplementary table 2. Terpenoid metabolism gene profiling in different tissues and developmental stages**

Gene expression matrix of predicted terpenoid metabolism genes. Expression units are expressed in RPKM.
