# Supplementary material for: In silico discovery of terpenoid metabolism in Cannabis sativa
Source: F1000Res. 2017 Feb 6;6:107. [Version 1] doi: 10.12688/f1000research.10778.1 (PMC5482328; doi:10.12688/f1000research.10778.1)
Supplement: Peer review history

# Peer review: Referee response for version 1

**DOI:** 10.5256/f1000research.11622.r23344

**Published:** 2017-06-19

**Authors:** Meirong Jia

**Affiliations:** 1 Roy J. Carver Department of Biochemistry, Biophysics & Molecular Biology, Iowa State University, Ames, IA, USA

The bioinformatics study here has reported 215 genes that are putatively involved in terpenoid biosynthesis in *Cannabis sativa *using available genome and transcriptome resources. This knowledge would predictably accelerate the terpenoid investigations in this specific species, or even broader. The research approaches are clear and sound. The writing is generally logical and organized. So I would recommend the publication of this work as a data note.

 I would suggest the author to make the following minor changes.

 1.  The author might wish to clarify throughout the text as to the original “transcriptome” (or “genome”) resources they have used for initial study. For instance, adding the specific link of that file name.

 2.  In the caption of figure 3, the author claimed “five gene clusters were defined” while in the text, it was “four gene clusters”, the author needs to correct this. Also, it is confused here that some genes clustered are expressed in “Finola” because from the previous text in “introduction” “para 3”, it was said the study was conducted “within the transcripts of the canSat3 reference transcriptome of Cannabis sativa21”. Please clarify this.

 3. The 215 gene candidates for terpenoids have been further predicted to express distinctively in tissues and developmental stages. While it is not easy to specifically check the expression pattern of each gene from the currently provided data. It would be great if possible that the author could add this piece of information, which would be valuable for future characterization of these genes. An example study showing the expression pattern of terpene genes would be found in the paper by Wang, *et al.* 2016 ^1^.

I have read this submission. I believe that I have an appropriate level of expertise to confirm that it is of an acceptable scientific standard.

## References

[1] WangQJiaMHuhJHMuchlinskiAPetersRJThollD: Identification of a Dolabellane Type Diterpene Synthase and other Root-Expressed Diterpene Synthases in Arabidopsis. *Front Plant Sci*.2016;7: 10.3389/fpls.2016.01761 1761 10.3389/fpls.2016.01761 27933080PMC5122590

# Peer review: Referee response for version 1

**DOI:** 10.5256/f1000research.11622.r23373

**Published:** 2017-06-09

**Authors:** Jonathan E Page, Judith K. Booth

**Affiliations:** 1 Botany Department, University of British Columbia, Vancouver, BC, Canada; 2 Michael Smith Laboratories, University of British Columbia, Vancouver, BC, Canada

The present study is appropriate as a data note, and may provide a useful shortcut for future studies on terpenoid biosynthesis in cannabis. The author identifies transcripts putatively involved in terpenoid biosynthesis, including processes of both primary and specialized metabolism. The rationale is well explained, and the methods are technically sound.

 Major changes:

  

- More information is required in the Materials and Methods for the study to be replicable. Two versions of the canSat3 transcriptome are available, and the authors should specify whether they used the full or representative transcript set. The authors must also provide the NCBI accession numbers for the GEO dataset they used.
- There is an unsupported statement in the results section, which reads: “Similar to other biological compounds, their abundance directly correlates with the expression levels of the enzymes involved in their metabolism.” In a complex system, this is not obvious and requires textual support.

 

 Minor changes:

  

- The Introduction generally provides a good rationale for the study. However, the definitions provided for ‘terpene’ and ‘terpenoid’ in the second sentence of the second paragraph do not reflect the definitions used in reference 12, nor are they the traditional definitions for those terms.
- References 24 and 25 address enzymes characterized in other organisms, and so do not support the statement regarding their biosynthetic activities in cannabis. That information is provided in reference 23.
- The final statement of the second introductory paragraph is unsupported and requires citation.
- Figure 2 caption states that the transcripts were taken from the reference genome, but the Materials and Methods indicate that a transcriptome was used.
- Figure 3 caption insufficiently explains x axis labels. The caption should define ‘PK’ and ‘Finola’. Otherwise, this figure shows an interesting and valuable result.

Readers should note the specific products of terpene synthases and other enzymes in specialized metabolism are currently difficult to predict using *in silico* methods. A recent paper (Booth et al., 2017 ^1^) includes biochemical and phylogenetic analysis of some of the candidate genes highlighted here, and may be of interest to readers.

We have read this submission. We believe that we have an appropriate level of expertise to confirm that it is of an acceptable scientific standard, however we have significant reservations, as outlined above.

## References

[1] BoothJKPageJEBohlmannJ: Terpene synthases from Cannabis sativa. *PLoS One*.2017;12(3) : 10.1371/journal.pone.0173911 e0173911 10.1371/journal.pone.0173911 28355238PMC5371325

# Peer review: Referee response for version 1

**DOI:** 10.5256/f1000research.11622.r19961

**Published:** 2017-03-10

**Authors:** Akan Das

**Affiliations:** 1 Department of Applied Biology, University of Science and Technology, Meghalaya (USTM), Ri-Bhoi, Meghalaya, India

In the present work, 23,630 transcripts were scanned for evolutionarily conserved protein domains and annotated using Gene Ontology analysis. A total of 215 evolutionarily conserved genes encoding enzymes presumably involved in terpenoid metabolism are described on the basis of Gene Expression profiles of NCBI GEO repository. The identification of candidate genes on terpenoid metabolism in *Cannabis sativa *will ease the future investigations on terpenoid metabolism pathways.

 It is a small piece of work on bioinformatics analysis of transcripts which present useful information to the plant researcher. Hence, the article can be recommended to be published as a Data Note. I suggest the author make a minor change in the title to be " *In silico* discovery of terpenoid metabolism associated transcripts in *Cannabis sativa" *and that the discussion, rather than describing general things, should be modified to discuss the findings in the work.

I have read this submission. I believe that I have an appropriate level of expertise to confirm that it is of an acceptable scientific standard.
